# eRMSF: A Python Package for Ensemble-Based RMSF Analysis of Biomolecular Systems

**DOI:** 10.1021/acs.jcim.5c02413

**Published:** 2025-11-19

**Authors:** Pablo Ricardo Arantes, Rodrigo Ligabue-Braun, Conrado Pedebos

**Affiliations:** Graduate Program in Biosciences (PPG Bio), 117303 Universidade Federal de Ciências da Saúde de Porto Alegre (UFCSPA); Rua Sarmento Leite, 245 - Centro Histórico, Porto Alegre 90050-170, Brasil

## Abstract

Understanding molecular flexibility and dynamics across different structural ensembles is essential for interpreting the behavior of complex biological systems. Here, we introduce eRMSF, a fast and user-friendly Python package built with MDAKit from MDAnalysis, designed to perform ensemble-based root mean square fluctuation (RMSF) analyses. Unlike traditional approaches limited to molecular dynamics trajectories, eRMSF extends flexibility analysis to ensembles generated by different methods, such as MD simulations, BioEmu (a deep learning tool for equilibrium ensemble prediction), subsampled AlphaFold2 (AlphaFold ensemble generation), and other computational or experimental sources. By enabling RMSF calculations across heterogeneous ensembles, eRMSF provides a unified framework to evaluate residue or atomic fluctuations in both simulated and predicted structures. Users can easily customize atom, residue, or region selections, tailoring analyses to specific research questions. This approach delivers high-resolution insights into localized motions, complements global stability assessments, and reveals dynamic regions often overlooked by single-method analyses. The repository for eRMSF is available at https://github.com/pablo-arantes/ermsfkit.

## Graphical abstract

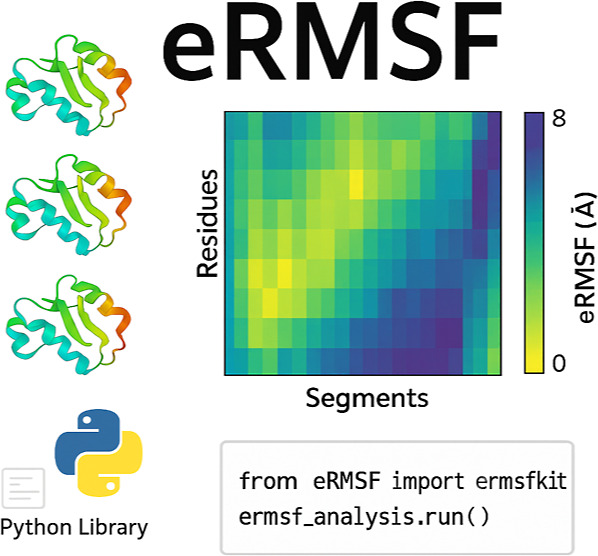

## Introduction

The study of biomolecular flexibility is central to understanding the structure–function relationship of proteins and nucleic acids. Molecular ensembles, which capture the range of conformations accessible to a system, provide a more realistic view of biomolecular dynamics than single static structures.^1–3^ Tools that allow researchers to rigorously analyze these ensembles in a fast and accessible way are essential for interpreting complex biological processes and for advancing drug discovery, protein engineering, and structural biology research.^4,5^ Accessibility is equally important, as intuitive and open-source tools empower a broader community of researchers to integrate ensemble-based analyses into their workflows.

Biomolecular ensembles can be generated through a variety of approaches, each with distinct advantages and limitations.^6^ Classical molecular dynamics (MD) simulations remain the gold standard for exploring conformational landscapes at atomistic resolution, but they are often computationally demanding.^2^ Complementary approaches, such as enhanced sampling techniques, can accelerate the exploration of rare conformations, while experimental techniques like NMR spectroscopy or cryo-EM also provide ensemble information. More recently, structure prediction methods such as AlphaFold2 ^7^ and RoseTTAFold^8^ have introduced strategies for generating multiple conformers by subsampling or perturbation, enabling the construction of structural ensembles at scale. This growing diversity of ensemble-generation strategies highlights the need for unified and flexible methods of analysis.

With the rise of deep learning–based approaches, such as BioEmu^9^ for equilibrium ensemble prediction and AlphaFold subsampling strategies for ensemble generation, the production of biomolecular ensembles has become faster and more accessible than ever. However, this shift also raises the critical question of how to evaluate the quality and reliability of these ensembles. New tools are required to bridge the gap between heterogeneous ensemble-generation methods and robust structural analysis. In this context, we present eRMSF, a Python package specifically designed to perform ensemble-based root mean square fluctuation (RMSF)^10^ calculations. By extending beyond traditional MD trajectories, eRMSF enables researchers to compare flexibility patterns across ensembles derived from diverse computational and experimental sources. While traditional RMSF relies on time-resolved trajectories, many modern structure generation methods, such as AlphaFold2, ESMFold, or diffusion, based AI models—produce ensembles of conformations without temporal information. The eRMSF formulation generalizes RMSF by quantifying positional variability across any set of structures, regardless of whether they originate from molecular dynamics or AI-based sampling. This enables direct comparison of flexibility between dynamic and static ensembles within a unified statistical framework. This makes it a versatile and powerful resource for systematically assessing local and global fluctuations, ultimately facilitating the integration of ensemble-based thinking into modern biomolecular research.

## Implementation

The eRMSF package was designed with flexibility and ease of use in mind, allowing researchers to analyze structural ensembles from a variety of sources. The tool accepts inputs derived from molecular dynamics (MD) simulations, AlphaFold2 subsampling ensembles, BioEmu-generated equilibrium ensembles, and other computational or experimental methods that provide structural models. By supporting this diversity of input types, eRMSF enables users to directly compare residue- or atom-level fluctuations across ensembles generated by fundamentally different approaches.

To accommodate the wide range of formats commonly used in structural biology, eRMSF is fully compatible with trajectory and structure file types supported by MDAnalysis,^11,12^ including DCD, XTC, PDB, and others. Users may input a single trajectory with an associated topology file, or a set of individual PDB structures representing an ensemble. This versatility ensures that ensembles generated by both simulation and prediction pipelines can be analyzed within the same framework.

At its core, eRMSF implements the standard definition of the root mean square fluctuation (RMSF), which measures the average positional deviation of an atom (or group of atoms) relative to its mean position across an ensemble. For an atom *i*, the RMSF is defined as

1 $$RMSF\left(i\right)=\sqrt{\frac{1}{N}\sum_{t=1}^{N}{∥r_{i}\left(t\right)-\left\langle r_{i}\right\rangle ∥}^{2}}$$

where **r**_*i*_(*t*) is the position of atom *i* at frame *t*, ⟨**r**_*i*_⟩ is the time- or ensemble-averaged position of the atom, and *N* is the number of frames or structures in the ensemble. In the context of ensemble RMSF (eRMSF), the summation extends over heterogeneous conformations derived from MD simulations, machine learning–based predictions, or experimental data, rather than being restricted to a single MD trajectory. This provides a consistent and quantitative measure of structural variability across different sources of ensembles.

In contrast to conventional RMSF, which measures deviations relative to the mean structure of the trajectory, eRMSF computes positional fluctuations relative to a fixed reference structure defined by the reference_frame parameter. This approach ensures that all segments or ensemble members are compared consistently, even when no temporal order exists (e.g., in AI-generated or NMR-derived ensembles).

For each atom (or residue) *i* and segment *s*, the ensemble-based RMSF is computed as

2 $${eRMSF}_{i,s}=\sqrt{\frac{1}{N_{s}}\sum_{j=1}^{N_{s}}{∥r_{i,j}^{\left(s\right)}-r_{i}^{ref}∥}^{2}}$$

where *N*_*s*_ is the number of frames in segment *s*, **r**_*i*,*j*_^(*s*)^ is the position of atom *i* in frame *j* of that segment, and **r**_*i*_^ref^ corresponds to the coordinates of atom *i* in the chosen global reference structure.

This formulation ensures that all positional deviations are calculated relative to a common reference frame, providing a consistent baseline across heterogeneous data sets. Consequently, eRMSF enables direct comparison of flexibility across segments or ensembles that may not share a continuous temporal relationship, such as AI-generated or experimentally derived conformations.

This segmentation strategy allows users to monitor how local flexibility evolves across the ensemble, providing a heatmap representation of fluctuations that highlights structural heterogeneity more effectively than a single averaged RMSF curve. The user controls the level of granularity through the skip parameter, which defines the number of frames per segment. For example, if an ensemble contains 100 frames and the user sets skip = 10, eRMSF partitions the trajectory into 10 segments of 10 frames each, yielding 10 fluctuation profiles along the *x*-axis. When skip = 1, each frame is treated as its own segment, maximizing temporal or ensemble resolution.

eRMSF can be run as a standalone Python script for automated batch analyses or integrated interactively into Jupyter notebooks,^13^ making it suitable for both high-throughput workflows and exploratory data analysis. For broader accessibility, the package also includes ready-to-use Google Colab^14,15^ notebooks (https://colab.research.google.com/github/pablo-arantes/ermsfkit/blob/main/eRMSF.ipynb), which allow users to perform ensemble RMSF analyses directly in the browser without requiring local installation or specialized hardware. These interactive notebooks provide step-by-step guidance, lowering the entry barrier for researchers with limited programming experience while remaining fully customizable for advanced users.

## eRMSF as a Python Library

In addition to being available as a standalone tool, eRMSF is implemented as a Python library within the MDAnalysis Toolkits (MDAKit)^16^ ecosystem, following the same modular architecture and design philosophy. This integration ensures that eRMSF inherits the flexibility, performance, and interoperability of the broader MDAnalysis toolkit, while providing a familiar interface for users already working with MDAKit-based workflows.

The core functionality of eRMSF is exposed through a set of Python functions and classes that mirror the conventions of MDAnalysis analysis modules. Users can define their system by providing a Universe object (topology and trajectory/ensemble files), followed by an atom or residue selection string (Figure 1). The main arguments closely follow MDAnalysis standards, including.

- Universe: an MDAnalysis Universe object containing the structural ensemble.
- Selection (default: “protein”): atom selection string to specify the subset of the system (e.g., backbone, side chains, custom residues).
- Start, stop, step: trajectory slicing options to control which frames are included in the analysis.
- Group by (optional): option to compute RMSF at the atom, residue, or region level.
- Output: choice of output format (e.g., NumPy arrays, Pandas DataFrames, or direct plotting).

**Figure 1:**
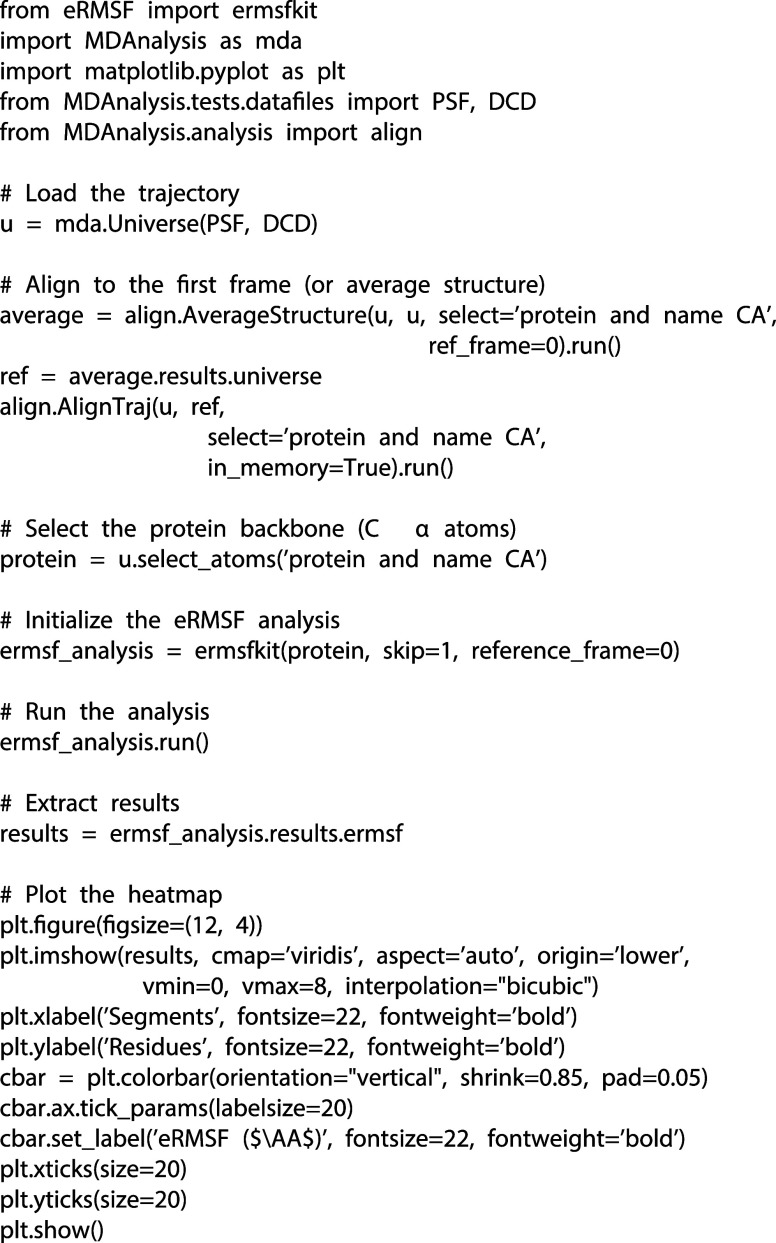
Minimal example of running eRMSF on a test trajectory using MDAnalysis. This example demonstrates how users can seamlessly integrate eRMSF into their MDAnalysis workflows, from trajectory alignment to analysis and visualization, with minimal additional code.

This design makes eRMSF immediately accessible to MDAnalysis users, allowing seamless integration with existing pipelines for structural analysis, visualization, and machine learning workflows. The results can be exported for downstream applications, such as comparing flexibility profiles across ensembles or integrating fluctuations into free energy and docking studies.

By adopting the MDAKit architecture, eRMSF ensures long-term maintainability and extensibility. Developers can easily extend the package to support new ensemble types or custom analysis metrics, while end users benefit from consistent syntax and interoperability across the MDAnalysis ecosystem.

## Usage

The eRMSF package can be used either as a standalone Python script or directly within a Jupyter notebook. Its interface follows the conventions of the MDAnalysis ecosystem, making it intuitive for researchers already familiar with trajectory and ensemble analysis. On Figure 1, we provide a minimal usage example that demonstrates the main steps of running an ensemble RMSF calculation.

The workflow can be summarized in the following steps.

1. Load the ensemble: create an MDAnalysis Universe from a trajectory/topology pair or from a set of PDB files.
2. Preprocess the system: align the trajectory to a reference structure (first frame or an average structure) to remove global translational and rotational motions.
3. Select atoms or residues: define the subset of the system to analyze (e.g., Cα atoms, backbone, side chains, or custom regions).
4. Initialize the eRMSF analysis: create an ermsfkit object, specifying the selection, reference frame, and the skip parameter that controls the segmentation of the ensemble.
5. Run the analysis: compute ensemble RMSF values for each segment of the trajectory or ensemble.
6. Retrieve and visualize results: the output is accessible as a NumPy array and can be plotted as a heatmap, where the *y*-axis corresponds to residues (or atoms) and the *x*-axis corresponds to segments defined by the skip value.

This design makes eRMSF straightforward to integrate into existing analysis pipelines while also providing interactive exploration options in Jupyter or Google Colab notebooks.

## Applications

To demonstrate the applicability of eRMSF, we tested the tool on three distinct structural ensembles of the Abl1 kinase. This protein was chosen because of its biological relevance in cancer and its extensive characterization in both experimental and computational studies. By applying eRMSF across ensembles generated from molecular dynamics simulations, BioEmu, and AlphaFold2 subsampling, we showcase its flexibility in analyzing fluctuations across heterogeneous sources of conformational data (Figure 2). In addition to Abl1 kinase, we also examined adenylate kinase (AdK), a classical model of large-scale conformational transitions, which is discussed in a dedicated subsection below. Similar approaches have been applied in previous studies,^5,17,18^ but those implementations were neither fully automated nor optimized for speed. In contrast, eRMSF provides a fast and automated framework, highlighting the utility of ensemble-based RMSF analyses for protein dynamics. Benchmark tests performed on Google Colab (2 CPU cores) show that the complete analysis of a 2000-frame trajectory of a 250-residue protein takes only about 11 s with skip = 1, demonstrating that eRMSF is highly efficient even on modest hardware without requiring explicit parallelization (Table S1).

**Figure 2:**
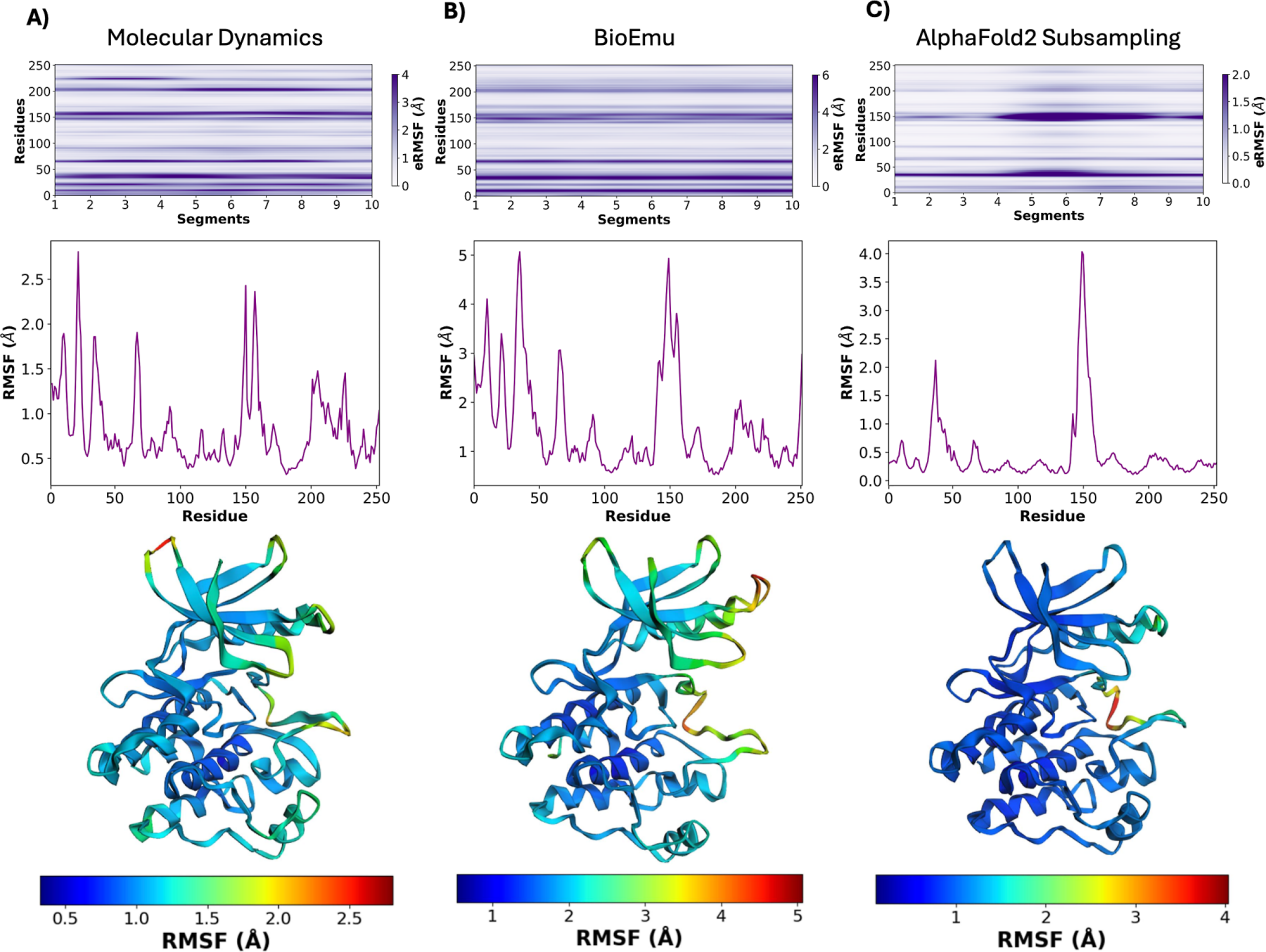
Application of eRMSF to different structural ensembles of Abl1 kinase. (A) Molecular dynamics (MD) ensemble, (B) BioEmu-predicted ensemble, and (C) AlphaFold2 subsampling ensemble. For each case, the top panel shows the eRMSF heatmap across residues and trajectory segments (generated using bicubic interpolation for smoother visualization), the middle panel displays the average RMSF per residue, and the bottom panel illustrates the corresponding protein structure colored according to residue fluctuations. While all three ensembles capture the flexible regions of Abl1, clear differences emerge in the magnitude and localization of fluctuations depending on the ensemble generation method.

### Molecular Dynamics Simulations

In the first application, we considered an ensemble of Abl1 kinase^19^ conformations generated through a conventional all-atom molecular dynamics (MD) simulation. The simulation captured the equilibrium dynamics of the protein, providing a dense trajectory of conformations sampled over time. eRMSF was applied to the trajectory in.dcd format, dividing it into segments to monitor residue-level fluctuations throughout the simulation. The resulting heatmap revealed pronounced flexibility in loop regions and domains associated with kinase activation, with fluctuations reaching up to ∼4 Å. This demonstrates the ability of eRMSF to identify dynamic hotspots and characterize the temporal distribution of flexibility within long MD trajectories.

### BioEmu Ensembles

The second application involved the use of BioEmu, a deep learning–based method for generating equilibrium ensembles of protein conformations. For Abl1 kinase, BioEmu generated an ensemble of diverse conformations without requiring explicit molecular simulations. Applying eRMSF to this data set produced fluctuation profiles consistent with functionally relevant regions identified in MD, validating the robustness of BioEmu-generated ensembles. Notably, the BioEmu ensemble displayed a narrower distribution of fluctuations than the MD trajectory, reflecting its equilibrium-based sampling strategy.

### AlphaFold2 Subsampling

Finally, we applied eRMSF to an ensemble generated through AlphaFold2 subsampling, where multiple structural models of Abl1 kinase were derived by varying input seeds and sampling strategies. Although these structures represent static predictions, their diversity captures alternative conformations relevant to protein flexibility. The resulting eRMSF profile revealed more restricted fluctuations (approximately 2 Å) compared to MD, particularly in flexible loops. This reflects the inherent biases of structure prediction models and highlights how eRMSF can expose the differences between simulated and predicted ensembles. Interestingly, the localized increase in fluctuation observed in the middle region of the AlphaFold2 subsampling ensemble corresponds to structural elements such as the activation loop and phosphate-binding loop, which are known to undergo large rearrangements between the active and inactive states of Abl1 kinase.^20^ This behavior reflects the intrinsic conformational heterogeneity captured by the subsampling approach, which perturbs MSA inputs to sample intermediate conformations spanning the transition between functional states. The observed fluctuations therefore highlight the sensitivity of eRMSF in detecting ensemble variability even among AI-generated structures lacking explicit temporal continuity.

### Comparative Insights

The comparison of eRMSF profiles across ensembles highlights both shared and distinct features of protein flexibility. For Abl1 kinase, the three ensembles capture similar regions of high and low fluctuations, supporting the robustness of the method in detecting conserved dynamic signatures. At the same time, differences in the magnitude and distribution of fluctuations reflect the specific sampling strategies underlying each ensemble. It is also important to note that the absolute scale of RMSF values differs between the ensembles, reflecting the distinct nature of the methods used to generate them. For example, the molecular dynamics ensemble reaches fluctuations of approximately 4 Å, while the AlphaFold2 subsampling ensemble shows more restricted fluctuations around 2 Å. These differences arise from the intrinsic variability captured by each approach and should be carefully considered when comparing ensembles. As shown in Figure S1, when all ensembles are plotted at the same scale, the BioEmu ensemble displays notably higher overall flexibility compared to MD and AF2 subsampling. This observation underscores the broader conformational diversity captured by BioEmu-generated ensembles and highlights the value of using a unified scale for direct visual comparison of fluctuation magnitudes. Taken together, these results underscore the potential of eRMSF to serve as a unifying framework for comparing ensembles derived from diverse sources.

### Detecting Conformational Transitions with eRMSF

In addition to ensemble comparisons, eRMSF is particularly valuable for analyzing trajectories that capture large-scale conformational changes, such as the closed-to-open transition of adenylate kinase (AdK),^21^ a phosphotransferase enzyme. In this case (Figure 3), the conformational transition occurs only after approximately 40% of the trajectory has elapsed. While conventional RMSF analysis confirms the presence of flexible regions, it cannot resolve when the transition initiates or how fluctuations evolve along the trajectory. By contrast, the segment-based approach of eRMSF makes it possible to localize the onset of structural rearrangements and track their progression, offering time-resolved insights into flexibility that are inaccessible with traditional RMSF calculations. This example reinforces the importance of eRMSF for dissecting dynamic events in biomolecular simulations, especially when conformational transitions are not uniformly distributed across the trajectory.

**Figure 3:**
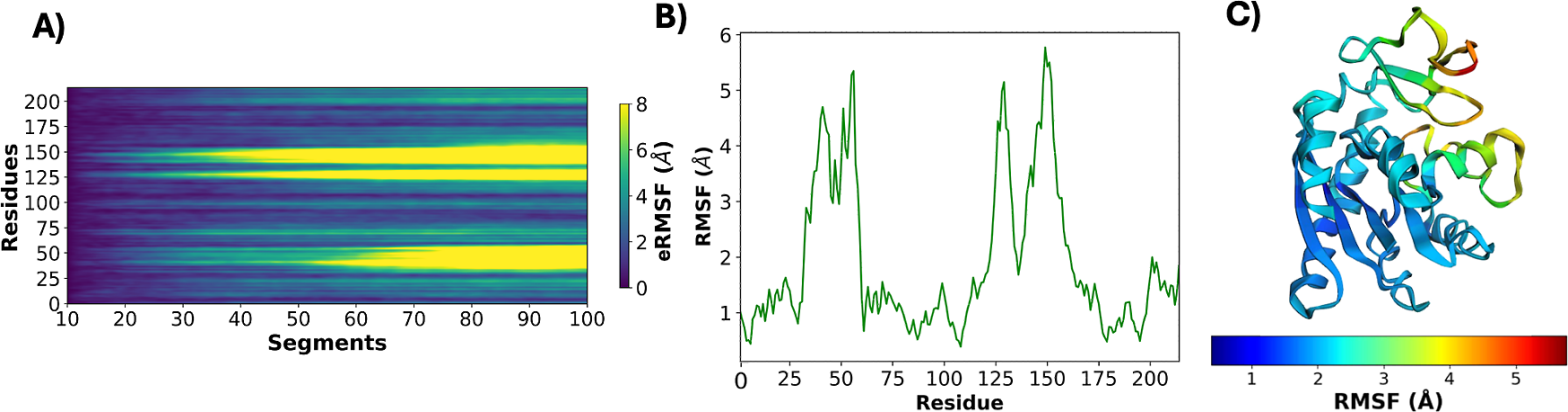
Application of eRMSF to the closed-to-open transition of adenylate kinase (AdK). (A) eRMSF heatmap across trajectory segments and residues (generated using bicubic interpolation for smoother visualization), (B) average RMSF profile per residue, and (C) structural representation of AdK colored by RMSF values. The eRMSF analysis reveals that the conformational transition occurs only after approximately 40% of the trajectory, highlighting the temporal localization of the structural rearrangement. In contrast, traditional RMSF calculations capture overall flexibility but cannot resolve when the transition starts or how it evolves along the trajectory. This demonstrates the ability of eRMSF to provide time-resolved insights into conformational dynamics.

## Conclusions

In this work, we presented eRMSF, a flexible and user-friendly tool for computing ensemble root-mean-square fluctuations across a wide range of structural data sources. By extending the conventional RMSF calculation to segmented ensembles, eRMSF provides a straightforward yet powerful way to capture and compare residue-level flexibility from molecular dynamics simulations, machine learning–generated ensembles such as BioEmu, and subsampled structural predictions from AlphaFold2. The eRMSF approach bridges the gap between time-dependent simulations and static AI-generated ensembles, offering a robust metric to compare structural heterogeneity across different sources of conformational sampling.

A key advantage of eRMSF lies in its simplicity and speed of use. The tool can be run either as a standalone Python script or within Jupyter notebooks, making it accessible to both novice and experienced users. The ability to easily adjust parameters such as the skip interval provides users with direct control over the resolution of the analysis, without compromising usability.

Integration of eRMSF into the MDAnalysis Toolkits (MDAKit) ecosystem ensures that the tool follows established community standards in molecular dynamics analysis. This not only facilitates adoption by a broad user base already familiar with the MDAnalysis infrastructure, but also strengthens reproducibility and interoperability across workflows. By lowering the barrier to analyzing structural ensembles, eRMSF contributes to advancing the field of computational biophysics, as intended with other strategies,^22–25^ enabling more systematic investigations of protein flexibility across heterogeneous data sets.

Overall, we expect that eRMSF will serve as a valuable addition to the toolkit of computational researchers, helping to bridge ensemble sources and providing deeper insights into protein dynamics in a fast, accessible, and reproducible manner.

## Supplementary Material



## Data Availability

The code presented here are freely and publicly available at: https://github.com/pablo-arantes/ermsfkit.

## Notes

All Supporting Information are available as separate files accompanying this manuscript. The Supporting Information is available free of charge at https://pubs.acs.org/doi/10.1021/acs.jcim.5c02413.

The Supporting Information provides complementary data and analyses that further illustrate the implementation and validation of the eRMSF method. Figure S1: Comparative eRMSF plots for the Abl1 ensembles obtained from BioEmu, Molecular Dynamics (MD) simulations, and AlphaFold2 subsampling, displayed on the same scale to enable direct comparison. The regions of highest flexibility are highlighted, showing that the BioEmu ensemble exhibits greater overall fluctuations compared to the other methods. Table S1: Benchmark of computational performance for eRMSF using a protein with approximately 250 residues and 2000 frames under different skip values, executed on Google Colab with two CPU cores. The results demonstrate that the implementation is highly efficient even without explicit parallelization (PDF)
