# Supplementary material for: eRMSF: A Python Package for Ensemble-Based RMSF Analysis of Biomolecular Systems
Source: J Chem Inf Model. 2025 Nov 19;65(23):12648–54. doi: 10.1021/acs.jcim.5c02413 (PMC12690589; doi:10.1021/acs.jcim.5c02413)
Supplement: Supplementary file 1 [file ci5c02413_si_001.pdf]

eRMSF: A Python Package for Ensemble-Based RMSF Analysis of Biomolecular Systems

Pablo Ricardo Arantes,* Rodrigo Ligabue-Braun, and Conrado Pedebos

Graduate Program in Biosciences (PPG Bio), Universidade Federal de Ciências da Saúde de Porto Alegre (UFCSPA); Rua Sarmento Leite, 245 - Centro Histórico, 90050-170 Porto Alegre, RS, Brasil

E-mail: pablitoarantes@gmail.com

Phone: +55 (19)993194366

Supporting Information

List of Figures

1 **Comparative eRMSF profiles across different ensembles.** eRMSF heatmaps for Abl1 kinase comparing flexibility profiles obtained from molecular dynamics (MD) simulations, BioEmu ensembles, and AlphaFold2 (AF2) subsampling. Residues 0–80 (left) and 120–250 (right) are shown for clarity. All ensembles were plotted using the same eRMSF scale to enable direct comparison of fluctuation magnitudes. When compared at the same scale, the BioEmu ensemble exhibits higher overall flexibility relative to the MD and AF2 ensembles, reflecting the broader conformational sampling achieved by BioEmu. Plots were generated using bicubic interpolation for smooth visualization. Regions of enhanced flexibility are highlighted by darker colors, illustrating both conserved and ensemble-specific fluctuation patterns. 4

Benchmark of Computational Performance

To assess the computational efficiency of `eRMSF`, we benchmarked the method using a protein system containing approximately 250 amino acid residues. The calculations were performed in a standard Google Colab environment equipped with two CPU cores. The benchmark results, summarized in Table S1, show that the analysis is extremely fast even without explicit parallelization, with the total execution time decreasing slightly as the trajectory frame skipping factor increases. These results reinforce that `eRMSF` provides a highly efficient implementation suitable for large-scale analyses of multiple ensembles.

Table 1: Benchmark results for a system of 250 residues using Google Colab (2 CPU cores). The analysis was performed with 2000 trajectory frames using different skip values.

**Number of Frames**	**Skip Value**	**Execution Time (s)**
2000	1	11
2000	2	9
2000	4	8
2000	6	7
2000	8	7
2000	10	6

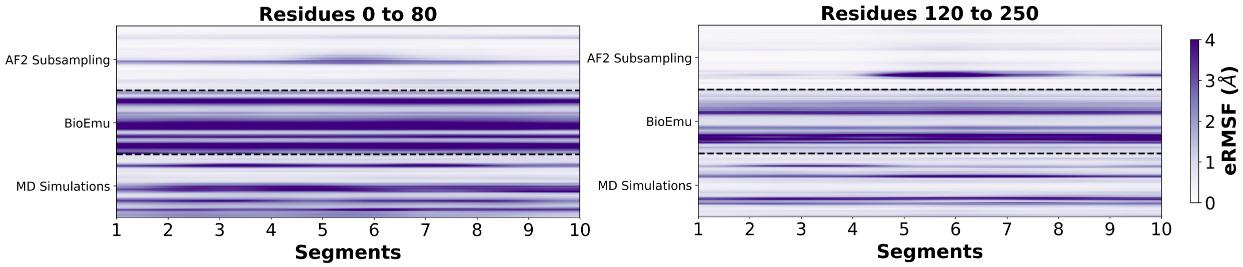

Figure S 1: **Comparative `eRMSF` profiles across different ensembles.** `eRMSF` heatmaps for Abl1 kinase comparing flexibility profiles obtained from molecular dynamics (MD) simulations, BioEmu ensembles, and AlphaFold2 (AF2) subsampling. Residues 0–80 (left) and 120–250 (right) are shown for clarity. All ensembles were plotted using the same `eRMSF` scale to enable direct comparison of fluctuation magnitudes. When compared at the same scale, the BioEmu ensemble exhibits higher overall flexibility relative to the MD and AF2 ensembles, reflecting the broader conformational sampling achieved by BioEmu. Plots were generated using bicubic interpolation for smooth visualization. Regions of enhanced flexibility are highlighted by darker colors, illustrating both conserved and ensemble-specific fluctuation patterns.
